# Mid-term outcomes of arthroscopic-assisted Core decompression of Precollapse osteonecrosis of femoral head—minimum of 5 year follow-up

**DOI:** 10.1186/s12891-019-2853-0

**Published:** 2019-10-15

**Authors:** Mark R. Nazal, Ali Parsa, Scott D. Martin

**Affiliations:** 1 Sports Medicine, Department of Orthopaedic Surgery, Massachusetts General Hospital, Partners Heath System, Boston, MA 02114 USA; 2 Orthopedic Research Center, Mashhad University of Medical Sciences (MUMS), Mashhad, Iran; 3 Department Of Orthopedic Surgery, Emam-Reza Hospital, Mashhad University of Medical Sciences, Mashhad, Iran

**Keywords:** Mid-term follow-up, Mid-term outcomes, Hip arthroscopy, Osteonecrosis, Osteonecrosis of the femoral head, ONFH, Core decompression, Mechanical symptoms, Femoral head preserving, Femoral head preservation

## Abstract

**Background:**

Osteonecrosis of the femoral head (ONFH) is a progressive disease that leads to collapse and the development of secondary arthritis. The preferred management of ONFH remains controversial. Arthroscopic-assisted management of ONFH is a new and evolving approach for hip preservation. We hypothesis that arthroscopy is able to improve ONFH outcomes by achieving accurate and minimally invasive decompression while successfully addressing concomitant intraarticular pathologies resulting in reliable mid-term outcomes.

**Methods:**

This was a retrospective cohort analysis. All patients had atraumatic ONFH with a precollapse lesion and a minimum follow-up of 5 years.

**Results:**

A total cohort of 11 hips (8 patients) was identified. The mean patient follow-up was 7 years ±1.48 years (range, 64—118 months). The Ficat-Alret classification found on preoperative imaging was Stage I—3 (27.2%), IIa—4 (36.4%), and IIb—4 (36.4%) hips. Four (36.4%) hips experienced mechanical issues, including locking, catching, and buckling. The most common concomitant pathology addressed at the time of arthroscopy, was labral repair/debridement—8 (73%), followed by microfracture—7 (64%). At final follow-up, 6 hips (54.5%) had not converted to THA. Upon further stratification, Stage I—100%, Stage IIa—75%, for a combined 87%, had not converted to THA, in contrast, 100% of hips categorized as Stage IIb had converted to THA. Ficat-Alret staging, especially Stage IIb, was significantly associated with conversion to THA. (*p*-value = 0.015) There were 0% major or minor complications.

**Conclusions:**

To our knowledge, this is the longest reported follow-up of arthroscopic-assisted management of ONFH. Arthroscopic-assisted management is a promising surgical approach that provides safe, accurate, and minimally invasive decompression, resulting in reliable results with an acceptable conversion rate to THA.

**Level of evidence:**

Level IV, Case Series.

## Background

Osteonecrosis of the femoral head (ONFH) is a progressive disease caused by an interruption of blood flow to the femoral head that leads to collapse and the development of secondary arthritis. Mont et al. estimated the prevalence of total hip arthroplasty (THA) involving osteonecrosis to be between 8—12%. With an estimated annual volume of THA of 371,000, it is projected that roughly 37,000 cases of THA involve ONFH [1–3]. Epidemiologically, patients in the 30’s to 40’s are commonly affected, resulting in a significant functional impairment in a relatively young population culminating in a loss of productivity for both the individual and society [4]. There are numerous direct and indirect risk factors associated with the development of ONFH. Direct factors include fractures, dislocation, sickle cell disease, and radiation. Indirect factors include corticosteroid use, excessive alcohol intake, certain metabolic syndromes, and smoking [3, 5]. Overall, the most commonly implicated risk factors are corticosteroids, alcohol, and trauma. Finally, it is also common for cases to occur idiopathically with no identified risk factor [6–8].

The preferred management of ONFH remains controversial and is largely dependent on the staging of the lesion. While there are several classification systems, the Ficat-Arlet staging is commonly used, with stages 0 through IIb considered precollapse, and stages III and IV considered collapsed [3, 9]. (Table 1) Management options can be categorized into observation, non-operative treatment, and operative treatment. Observation may allow small asymptomatic lesions to spontaneously resolve, although most lesions will progress [10]. Lieberman et al. found that 67% of asymptomatic hips and 85% of symptomatic hips managed with observation progress to collapse [11]. Non-operative therapies include bisphosphates, statins, prostaglandins, and hyperbaric oxygen. These treatments have been shown to decrease pain, but long-term outcomes are uncertain and require randomized control trials [10, 12]. Mont et al. literature review proposes that non-operative treatment may be attempted in asymptomatic hips, but once symptoms manifest operative treatment is indicated [7].

**Table 1:** Ficat-Alret Classification System

Stage	Symptoms	Radiograph Finding
0	–	Normal
I	Mild	Normal
IIa	Mild	Normal Head Contour, Sclerosis, Cysts
IIb	Moderate	Flattening of Femoral Head
III	Moderate to Severe	Collapse, Loss of Sphericity
IV	Severe	Acetabular Changes

Operative management is divided into two main categories: femoral head-preserving and arthroplasty [13]. Femoral head-preserving techniques have demonstrated successful outcomes in precollapsed staged lesions. Within the femoral head-preservation category there are numerous methods, variations, and adjuvants. The treatment algorithm for determining which method to pursue is largely based on staging and is still being delineated. Common head-preserving methods includes: 1) standard core decompression (CD), 2) multiple drilling decompression, 3) femoral rotational osteotomies, 4) non-vascularized bone graft, 5) vascularized bone graft, and 6) tantalum rod placement. Once collapse has occurred arthroplasty is indicated with excellent outcomes. Arthroplasty consists of either resurfacing or hip replacement [10, 12]. Ancelin et al. found similar THA survivability rates in patients with ONFH and primary osteoarthritis at ten-year follow-up [14].

Although there is no standard approach to the management of ONFH, CD is considered the most common treatment for early stage lesions. This management is based on the principle of decreasing intraosseous hypertension that is created by the necrosis process and inflammatory cell infiltration into the affected areas [10]. CD is performed percutaneously, using an 8—10 mm trephine that targets the area of necrosis using fluoroscopic guidance. Whereas, multiple drilling using a smaller diameter trephine to create more than one core track, and has achieved favorable outcomes while having lower complication rates, including subtrochanteric fracture [13, 15]. Al Omran et al. compared standard core decompression and multiple drilling in a cohort of patients with sickle cell disease, finding no statistically significance in outcomes or complications, although the multiple drilling technique had the benefit of being less invasive [16].

Although the literature has considered the various surgical techniques, arthroscopic management of ONFH is a new and evolving approach for hip preservation. One of the earliest descriptions of arthroscopic management of ONFH was reported by Ruch et al. in which arthroscopy was used to document accurate positioning of the core decompression [17]. Since Ruch’s paper in 1998, arthroscopic management has developed a relatively limited presence in the literature: one review article by Papavasiliou et al., several technique papers, and very few cohort analyses [4, 18–23]. With this limited body of literature, the purpose of this study is to provide further evaluation of arthroscopic-assisted core depression in the treatment of patients with ONFH, while being the first study to attain mid-term follow-up. We hypothesize that this cohort analysis will demonstrate that hip arthroscopy is able to treat ONFH by achieving accurate and minimally invasive decompression; in addition to successfully addressing concomitant intraarticular pathologies resulting in reliable mid-term survivability and an acceptable rate of conversion to THA.

## Methods

### Study design

This was a retrospective cohort analysis to evaluate the mid-term results of the arthroscopic management of ONFH. After obtaining Institutional Review Board (IRB) approval, all patients who underwent hip arthroscopy between June 2007 and June 2013 by the senior author at a hip-preserving service of a tertiary hospital were identified. A sub-cohort of patients diagnosed with non-traumatic ONFH were subsequently identified. All patients were assessed by the senior author and received a standard preoperative workup for patients undergoing a hip arthroscopy, including standard hip radiographs and hip MRI to evaluate the extent of disease and assess for possible quiescent ONFH in the contralateral hip. Based on hip radiographs, hip MRI, and clinical presentation, each hip’s ONFH was staged using the Ficat-Alret system [9]. (Table 1) The Ficat-Alret was selected, as opposed to other classification systems such as the Steinberg system, because it is routinely used by the MSK radiologists at our institution and because most of the literature on ONFH utilizes this system allowing for easier comparison of results.

In addition, preoperative imaging allowed for the diagnosis of concomitant hip pathologies, including labral tear, cam lesions, pincer lesions, and cartilage flaps. However, concomitant pathology did not affect the decision to perform hip arthroscopy, as the primary indication for surgery was treatment of the ONFH. Furthermore, additional workup of intraarticular pathology was, such as an intra-articular injection, was not performed.

Inclusion criteria consisted of hip arthroscopy for treatment of ONFH, patients with radiographic findings of Stage 0, I, IIa, and IIB (precollapse) based on Ficat-Alret classification for OFNH, and follow-up of a minimum of 5-years. Exclusion criteria were: traumatic ONFH, Stage III or greater severity based on the Ficat-Alret classification, dysplastic hips, or complication of consumptive disease.

### Patient selection

**Fig. 1:**
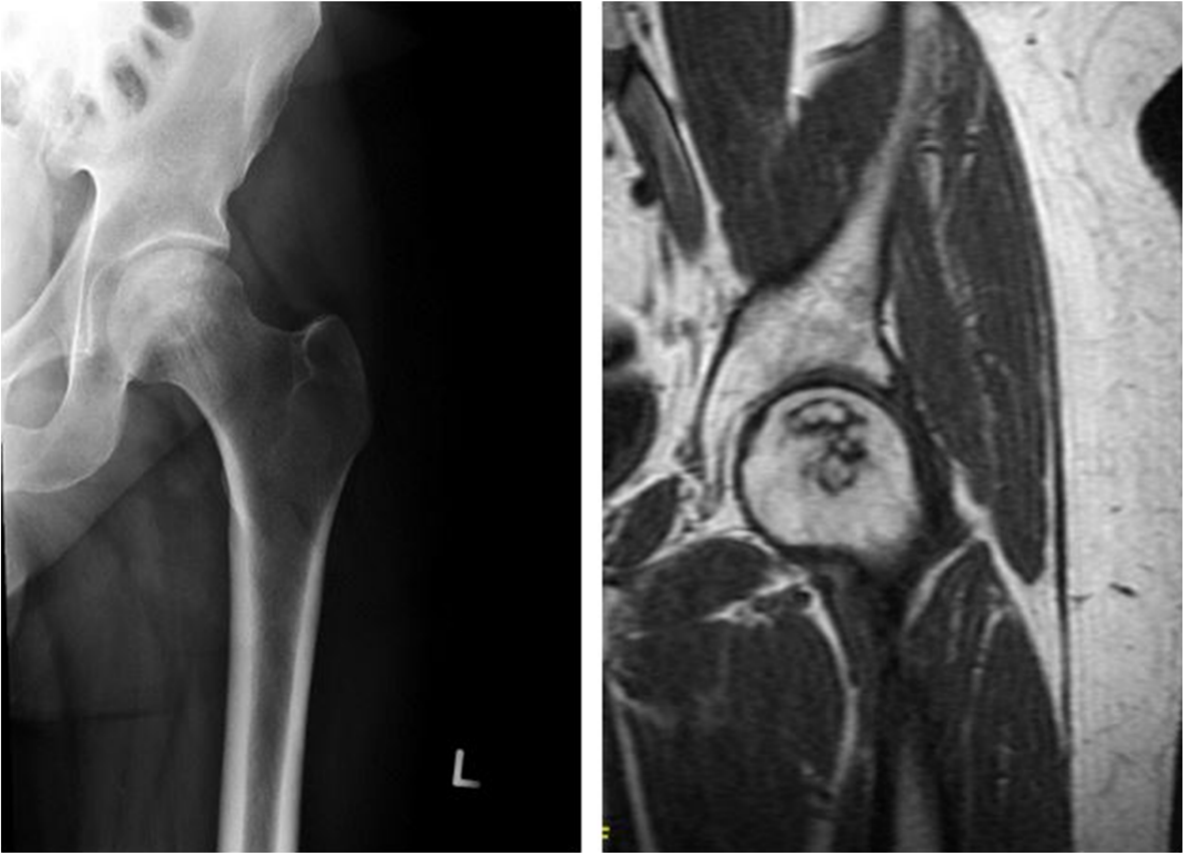
**a** Preoperative plain AP X-ray of left hip demonstrating a Stage IIa osteonecrotic lesion. **b** MRI T1-weighted coronal image of left hip shows a geographic pattern of the necrotic area

Standard radiographs and 1.5 T MRI of both hips were used to confirm the diagnosis and determine staging (Fig. 1). Among the 541 records of hip arthroscopy performed by senior author between July 2007 and July 2013, eleven hips in eight patients were identified with a documented or radiographic diagnosis of avascular necrosis. No hips were excluded as all 11 met the aforementioned criteria. Each patient’s preoperative history, the onset and duration of hip symptoms, physical examination, plain radiographs, MRI imaging, and operative notes were reviewed from a prospectively-derived database.

### Surgical intervention

All patients were operated on by the senior author. Each patient was given general anesthesia with endotracheal tube intubation. The patients were placed in the standard supine position on a hip arthroscopy table against a well-padded perineal post. Both feet are placed in protective foam boots and secured in the Smith & Nephew supine hip distractor apparatus. Our standard practice is to utilize intermittent traction when preforming central compartment work [24]. This decreases the risk of nerve injury and minimizes further stress or advancement of the disruption to the terminal circulation of the femoral head. A 17-gauge needle with a nitinol wire was used to establish the anterolateral portal under C-arm image intensifier guidance. The rest of the portals (anterior, mid-anterior and dienst) were established under direct arthroscopic visualization, with four portals being utilized in all patients. A pressure regulated flow pump was utilized to keep the arthroscopic pressure at 40 mmHg. (GoFlo Pump, Smith & Nephew, Andover, MA) Similar to utilizing gentle traction, the pressure was kept as low as possible compression and disruption to the terminal circulation of the femoral head.

An arthroscopic examination of the hip joint was performed, including: assessment of the femoral head for the presence of collapse, assessment of the articular cartilage overlying the acetabulum, probing of the articular cartilage to identify any softening or chondral flaps, and inspection of the condition of chondrolabral junction. If a labral tear was present, it was addressed either by labral fixation or debridement. Synovectomy was also performed if significant synovitis was observed.

**Fig. 2:**
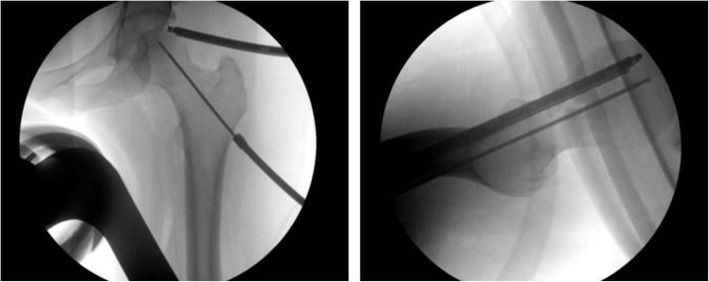
**a** Fluoroscopy AP of left hip demonstrating the positioning of the guide wire in the femoral head directed towards the necrotic area, while monitoring the femoral head cartilage by arthroscope to avoid articular cartilage penetration. **b** Fluoroscopy Lateral of left hip showing the positioning of the guide wire

Fluoroscopy was utilized to supplement the probing of the femoral head to assess the necrotic area for chondral softening and lack of subchondral support. A 1—2 cm incision was made on lateral proximal thigh through which a guiding pin was introduced and directed towards the necrotic lesion with direct fluoroscopic guidance (Fig. 2). Placement and trajectory of the guide pin was verified on both the AP and lateral views and arthroscopically by visualization of the pin’s advancement into the area of chondral softening. Then a 9-mm cannulated reamer was inserted over the guide pin, placement was again verified by fluoroscopy and arthroscopic visualization, after which core decompression was performed. The target of the reamer was the center of the necrotic area, while keeping at least a 3 mm distance to the subchondral bone. This safety distance, along with visualization by arthroscopy, was to ensure that there was no perforation of the cortical bone, articular cartilage, or joint space by either the guide pin or the reamer. After completion of the procedure, the arthroscope and tools were removed, and the portals were closed in a routine manner.

### Post-operative rehabilitation and follow-up

Post-operatively, patients were instructed to ambulate using crutches with a flat-foot gait for protected weight-bearing for 6—8 weeks. At six weeks, patients could get onto a stationary bike without utilizing resistance to focus on preserving and increasing their range of movement. At ten weeks, patients advanced to exercising with an elliptical trainer without utilizing resistance, with advancement to full weight-bearing at 10—12 weeks. Muscle strengthening began at four months, limited to short arc leg presses and reverse hamstring curls. Patients were released from physical activity limitations at six months, with instructions to return to activities gradually. Patients had scheduled follow-up visits at 4, 8, 12-weeks, and then every 6 months. Post-operative X-rays were obtained at the 8-week visit to evaluate the condition of the femoral head. Post-operative MRI was not routinely obtained.

Data points were extracted by chart review of the electronic medical record. The primary outcome was conversion to THA after hip arthroscopy-assisted management of ONFH. In addition, the time interval from arthroscopy to THA was calculated. Association of conversion to THA with Ficat-Alret classification, presence of mechanical symptoms, risk factors of ONFH, or bilateral hip involvement, was examined. Secondary data points include patient demographics (age, gender, BMI, laterality, center-edge angle), symptomatic interval duration before hip arthroscopy, arthroscopy findings and procedures performed during each surgery, postoperative VAS (Visual Analogue Scale) pain scale at latest follow-up, and the length of follow-up. Finally, complications were evaluated, including both major complications, such as sub-trochanteric femur fracture, violation of articular cartilage, extra-articular fluid extravasation, hip dislocation, thromboembolism, and septic joint; and minor complications, including adhesions, neurapraxia, broken instrumentation, superficial wound infection, and heterotopic ossification.

### Statistical analysis

Statistical analysis was performed using R Statistical software version 3.5.2 (Foundation for Statistical Computing, Vienna, Austria). Categorical statistics were represented as a number and percentage, while continuous statistics were represented as a mean average and standard deviation. Statistical testing was conducted using Fisher’s Exact Test for correlation of categorical variables. A *p*-value less than 0.05 indicated a significant difference.

## Results

A total cohort of 11 hips (8 patients) was identified, of which 1 patient (12.5%) was female and 7 patients (87.5%) were male. All eight patients were included based on the aforementioned inclusion and exclusion criteria. The mean age of the cohort was 36.4 years ±9.2 years (range, 17–48). (Table 2) The mean center-edge angle was 30.5° ± 3. 4° (range 26°— 36°). Patients’ past medical history was evaluated for established risk factors for the development to ONFH. Two hips (18.2%) had inhaled corticosteroid exposure and 4 hips (36.4%) had a history of opioid abuse. (Table 3).

**Table 2:** Demographic Characteristics

Mean Age, years ± SD (range)	36.4 ± 9.2 (16.9–47.6)
Gender, n (%)	
Male	7 (87.5%)
Female	1 (12.5%)
BMI, BMI ± SD (range)	26.7 ± 3 (21—31)
Patients Treated Bilaterally, n (%)	3 (27.3%)
Laterality, n (%)	
Left	7 (63.6%)
Right	4 (36.4%)
Mechanical Symptoms, n (%)	4 (36.4%)
Center-Edge Angle (CEA)	30.5° ± 3.4° (26°—36°)
Mean Follow-up, months ± SD (range)	84.7 ± 21.2 (64—118)
Pre-operative Symptom Duration, months ± SD (range)	14.5 ± 9.04 (4—36)

*BMI* Body Mass Index in kg/m^2^. Mechanical symptoms include locking, catching, and buckling. Pre-operative symptom duration is the time interval from when the patient began experiencing hip pain to when they are underwent hip arthroscopy

**Table 3:** Risk Factors of ONFH and Conversion to THA

Patient	Hip	Inhaled Corticosteroid	Smoking Tobacco	Excessive Alcohol Intake	Conversion to THA
1	1	–	–	–	Yes
2 (BL)	2	–	Yes	–	–
	3	–	Yes	–	–
3	4	–	–	–	Yes
4	5	–	–	–	–
5	6	–	–	–	–
6 (BL)	7	Yes	Yes	–	Yes
	8	Yes	Yes	–	Yes
7 (BL)	9	–	–	–	–
	10	–	–	–	–
8	11	–	–	–	Yes
n (%)		2 (18.2%)	4 (36.4%)	0 (0.00%)	5 (45.5%)

The Ficat-Alret classification found on preoperative imaging was Stage I in 3 (27.2%), IIa in 4 (36.4%), and IIb in 4 (36.4%) hips. (Table 4) The mean preoperative symptom duration from onset of symptoms to arthroscopic intervention was 14.5 months ±9.04 months (range, 4—36 months). Four (36.4%) hips experienced mechanical issues, including locking, catching, or buckling. The most common concomitant pathology addressed at the time of arthroscopy, was labral repair/debridement, followed by microfracture. (Table 4) The mean traction time was 56.7 min ± 11.4 min (range, 43—78) and mean operative time was 100.9 min ± 16.6 min (range, 85—130). Arthroscopic verification of guide pin and reamer placement was achieved in all 11 surgeries. Finally, there were no major complications (0%), including sub-trochanteric fracture or violation of the articular cartilage; or minor complications.

**Table 4:** Case Series Patient Details: is in Landscape page layout, and has been uploaded separately

Patient	Hip	Age Range (yr)	Gender	Laterality	Preop Ficat-Alret Staging	Mechanical Symptoms	Kerboul Angle:	Arthroscopy Findings	Arthroscopy Procedure	Postop VAS Score	Complications Major/Minor	Conversion to THA	Time from Arthroscopy to THA (mo)
1	1	40—45	M	R	IIb	Popping	80.2°	Cartilage Wear, Synovitis	Microfracture, Synovectomy	1	−−/−−	Yes	7
2 (BL)	2	30—35	M	R	IIa	–	101.7°	Chondral Defect, Labral Tear	Labral Debridement	1	−−/−−	–	–
	3	30—35	M	L	I	–	154.6°	Cartilage Wear, Labral Tear	Labral Debridement	1	−−/−−	–	–
3	4	40—45	M	L	IIa	–	96.6°	Cam Lesion, Pincer Lesion, Labral Tear	Femoral and Acetabular Osteoplasty, Labral Repair	1	−−/−−	Yes	33
4	5	15—20	M	L	I	Popping	80.9°	Pincer Lesion, Labral Tear	Acetabular Osteoplasty, Labral Repair	0	−−/−−		–
5	6	40—45	F	L	IIa	–	107.5°	–	–	0	−−/−−	–	–
6 (BL)	7	46—50	M	R	IIb	–	108.0°	Cartilage Wear, Synovitis	Microfracture, Synovectomy	1	−−/−−	Yes	23
	8	46—50	M	L	IIb	–	176.5°	–	–	1	−−/−−	Yes	25
7 (BL)	9	30—35	M	L	I	Clicking	104.2°	Cartilage Wear, Chondral Defect	Microfracture	1	−−/−−	–	–
	10	30—35	M	R	IIa	Clicking	106.1°	Cartilage Wear	Microfracture	1	−−/−−	–	–
8	11	36—40	M	L	IIb	–	187.0°	Chondral defect	Microfracture	1	−−/−−	Yes	25

Age Range, instead of specific age, is provided to help ensure patient confidentiality

*BL* Bilateral

Gender: *M* Male, *F* Female

Laterality: *L* Left, *R* Right

Kerboul Angle: combined necrotic angle or summation of angle of necrotic area seen on AP and lateral radiographs; small ≤160°, medium: 161–199°, large ≥200°

Major complications: sub-trochanteric fracture, violation of articular cartilage, extra-articular fluid extravasation, hip dislocation, thromboembolism, and septic joint

Minor complications: adhesions, neurapraxia, broken instrumentation, superficial wound infection, and heterotopic ossification

The primary outcome of this study was conversion to THA. The mean patient follow-up was 7 years ±1.48 years (range, 64—118 months). At final follow-up, 6 hips (54.5%) had not converted to THA. Upon further stratification, Stage I—100%, Stage IIa—75%, for a combined 87%, had not converted to THA, in contrast, 100% of hips categorized as Stage IIb had converted to THA. (Table 5) The indication for all five conversions to THA was symptomatic and radiographic progression of the ONFH. Of the five hips that converted to THA, the time interval from hip arthroscopy to THA was 22.6 months ±9.5 months (range, 7—33 months).

**Table 5:** Conversion to THA

	Conversion to THA	No THA	*p*-value
Mechanical Symptoms	1/4	3/4	0.55
Ficat-Alret Stage:			0.03*
Stage I	0/3	3/3	0.18
Stage IIa	1/4	3/4	0.55
Stage IIb	4/4	0/4	0.015*
Risk Factors			
Inhaled Corticosteroid	2/2	0/2	0.18
Smoking Tobacco	2/4	2/4	1.00
Bilateral Hip Involvement	2/6	4/6	0.57

Mechanical symptoms include locking, catching, and buckling. Statistical testing was conducted using Fisher’s Exact Test for correlation of categorical variables. A *p*-value less than 0.05 indicated a significant difference

Conversion to THA was analyzed for correlation with other variables and was found to be associated with Ficat-Alret staging of the hip. (*p*-value = 0.03) (Table 5) Further analysis found that this correlation was particularly significant for the Stage IIb subset of hips. (p-value = 0.015) The correlation of conversion to THA with mechanical symptoms, inhaled corticosteroid exposure, smoking tobacco exposure, and bilateral hip involvement of ONFH, was not statistically significant.

## Discussion

**Fig. 3:**
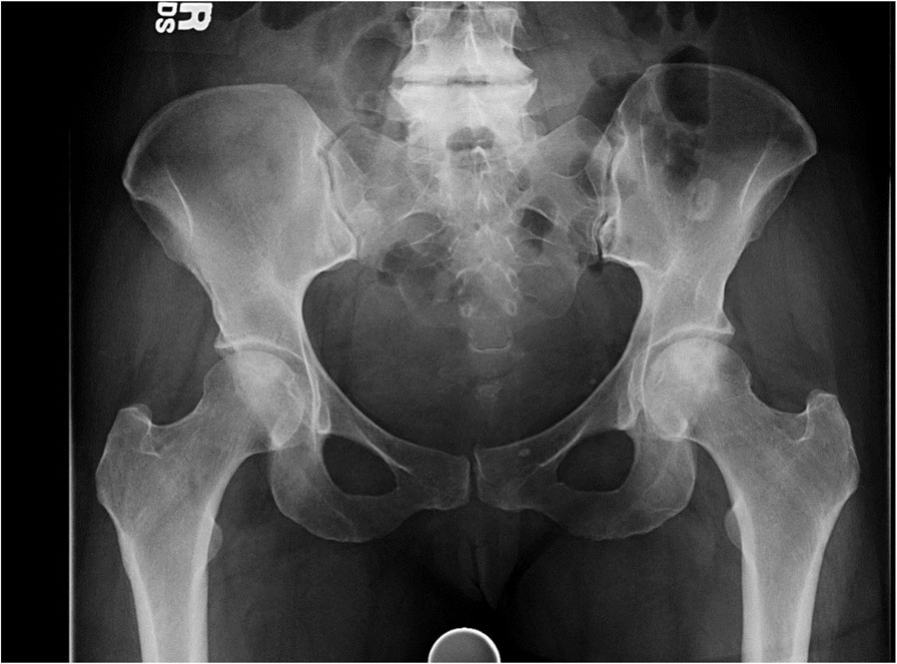
Plain AP pelvis X-ray at 5 years follow-up of a patient with a Stage IIa lesion of the left hip that shows preserved joint space without lesion progression

This study demonstrated good survivability of arthroscopic-assisted standard core decompression (CD) in precollapse ONFH with no complications at mid-term follow-up. In this cohort of 11 hips with an average follow-up of 7 years (with a minimum of 5 years), 6 patients (54.5%) had not converted to THA. (Fig. 3) However, when stratified by lesion stage, 100% of Stage I hips, 75% of Stage IIa, and 0% of Stage IIb hips had not converted to THA.

This study’s survivability is consistent with recently published data. Mukisi-Makaza et al. reported survivability in sickle cell patients with an average follow-up of 7.4 years utilizing the Ficat classification: 100% for Stage I and 47% for Stage IIa (did not report the conversion rate in Stage IIb lesions) [25]. Yang et al. reported similar rates using the Steinberg classification: 61% for Stage II and 25% in Stage III [26]. Another study with shorter follow-up of 3 years, evaluated CD with physical therapy reported similar rates for early lesions: 90% for Stage I and 82% for Stage II [27]. Finally, utilizing multiple drilling, Al Omran et al. reported similar survivability rates at a mean follow-up of 6.1 years: 83% for Stage I, 79% for Stage IIa, and 69% for Stage IIb [28].

Several studies have found that CD is more effective in the treatment of early avascular necrosis in the precollapse stage, particularly when the lesion is small (Keboul Necrotic Angle < 160°) or medium-sized (Keboul Necrotic Angle between 161—199°) [7]. This is congruent with this study finding high survivability for Stages I and IIa, but poor survivability for Stage IIb. Although this study has a small sample size, these findings indicate that arthroscopic-assisted management should be focused on earlier staged ONFH.

In this cohort, there were no (0%) major or minor complications, including femoral fracture or violation of the cartilage. Comparatively, percutaneous standard CD is associated with a 10—15% complication rate, including weakening the bone contributing to collapse, subtrochanteric fracture, or violation of the articular cartilage [7, 12]. Therefore, arthroscopic-assisted CD is associated with fewer complications [21].

Several variables were evaluated for correlation with conversion to THA. Ficat-Alret staging was found have a statistically significant association, particularly for Stage IIb lesions. This makes sense intuitively, as advanced lesions are likely to continue to progress to collapse. This may also indicate a decreased efficacy of treating advanced ONFH with arthroscopic-assisted decompression. Interestingly, ONFH of bilateral hip involvement, mechanical symptoms, inhaled corticosteroid and smoking tobacco exposure, where not significantly associated with conversion to THA. Although, one might expect mechanical symptoms to be associated with advanced ONFH and therefore more likely to be associated with conversion to THA, these symptoms can be due to numerous intra- and extraarticular etiologies [29]. The lack of statistical significant may be due to the small sample size of this study. Similarly, Bozic et al. reported advanced radiographic stage as a significant predictor of CD failure (defined as radiographic progression to Stage III or requiring a subsequent operation). In addition, that study reported shorter duration of symptoms and use of corticosteroids as significant predictors of failure, but no association with age, gender, alcohol intake, or renal transplantation [30].

**Fig. 4:**
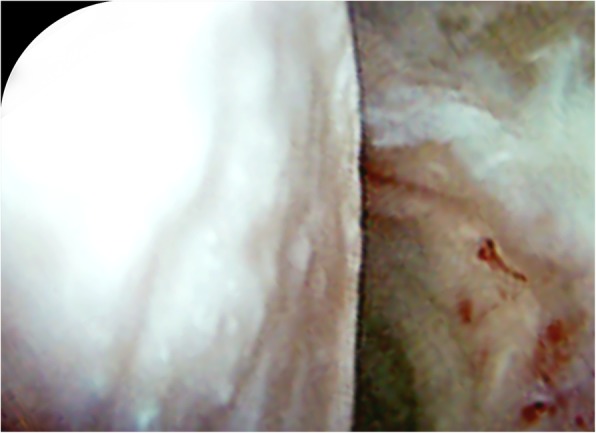
Intraoperative arthroscopic image demonstrating flattening of femoral head. Arthroscopy functions diagnostically, by allowing for the precise assessment of the articular surfaces of both the femoral head and the acetabulum for accurate staging and evaluation

Arthroscopic management of ONFH is advantageous because it is both diagnostic and therapeutic. Guadilla et al.’s report of the arthroscopic management of ONFH by CD with adjuvant platelet-rich plasma (PRP) described the advantages of arthroscopy as: diagnosis of concomitant pathologies, precise localization and drilling of necrotic lesions, and increased avoidance of joint penetration [18]. Diagnostically, arthroscopy allows for the precise assessment of the articular surfaces of both the femoral head and the acetabulum for accurate staging and evaluation. (Fig. 4) In this study, 6 hips (54.5%) had focal changes in the articular surface of the femoral head, while 2 hips (18.2%) had diffuse changes, with parallel results seen on the acetabular side.

**Fig. 5:**
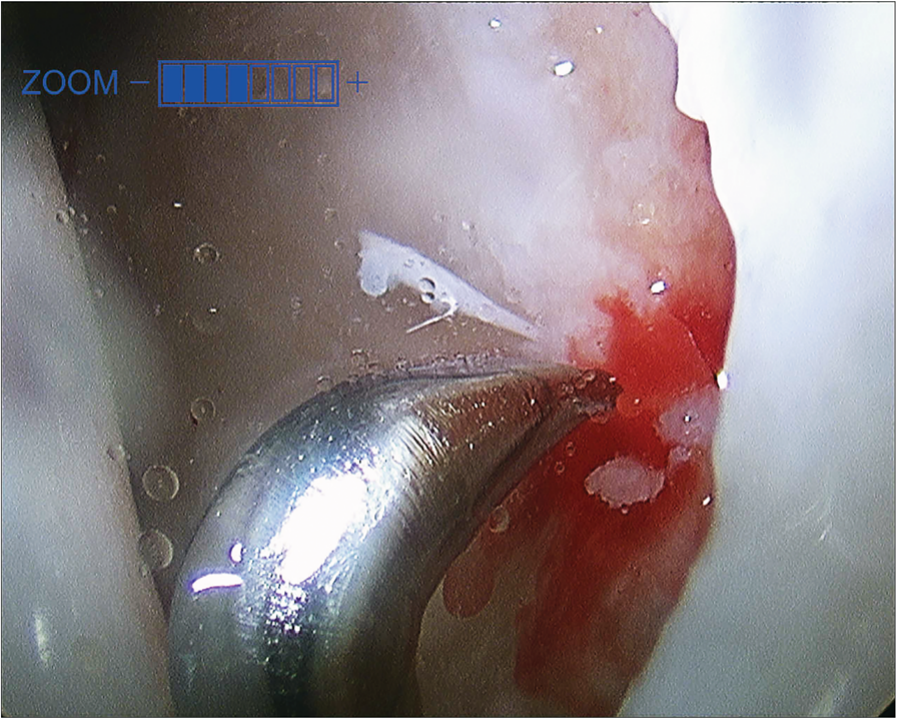
Intraoperative arthroscopic image demonstrating treatment of chondral wear with microfracture of the acetabulum. Arthroscopy functions therapeutically for the entire hip joint, by treatment of concomitant intra-articular pathology, including: loose bodies, pincer lesions, cam lesions, synovitis, labral tears, and chondral defects

Unlike percutaneous standard CD, arthroscopic-assisted CD allows for the arthroscopic treatment of concomitant intra-articular pathology associated with ONFH, including: loose bodies, pincer lesions, cam lesions, synovitis, labral tears, and chondral defects [20]. (Fig. 5) In our cohort, concomitant pathologies addressed includes: labral repair/debridement in 8 (73%), microfracture in 7 (64%), femoral osteochondroplasty in 1 (9%), and synovectomy in 1 (9%), hip(s), respectively. At latest follow-up, the concomitant pathologies had resolved with the 6 hips that did not convert to THA reporting little to no pain and unremarkable clinical exam.

Of the 11 hips, 4 (36.4%) had mechanical symptoms, all of which resolved after arthroscopic treatment. (Table 4) Even with lesions in the pre-collapse stage, some patients develop mechanical symptoms such as locking, buckling, catching, popping, or clicking. In this subset of patients, the mechanical issues warrant arthroscopic treatment. Because similar to the treatment of intra-articular pathologies, these mechanical issues are not addressed by percutaneous standard CD. McCarthy et al. performed hip arthroscopy on seven patients between 1993 and 2000 with documented or radiographically confirmed avascular necrosis [31]. This study concluded that hip arthroscopy is minimally invasive and an effective treatment for early ONFH with mechanical symptoms.

Interestingly, the effect of irrigation pressure, traction, and osteoplasty terminal circulation of the femoral head is not known. Theoretically, traction or irrigation pressure could compress the terminal circulation of the femoral head, resulting in worsening of the underlying pathology of ONFH. And osteoplasty of the femoral neck in the treatment of CAM lesions could expose the trabecular bone to the pressured irrigation fluid further compressing terminal circulation. However, our practice is to utilize intermittent traction only when working in the central compartment and to use minimal irrigation pressure (pressure controlled at 40 mmHg). Furthermore, the traction and irrigation pressure are temporary factors (mean traction time of 57 min and mean operative time of 101 min), whereas CD would decrease intraosseous pressure for the future of the native hip.

Although both CD and multiple drilling decompression are used to treat ONFH, this study chose to utilize CD because it is the most common and fundamental femoral-head preserving modality. It also has the benefits of technical ease and efficiency. Unfortunately, regardless of technique, even when femoral head-preserving surgery is attempted in the early stages of ONFH a substantial portion of patients continue to progress to THA, with variable levels and durations of survivability [7]. In this study with an average follow-up of 7 years, 13% of lesions in Stage I or IIa converted to THA, while a 100% of lesions in Stage IIb converted to THA. This supports the treatment principles that head-preservation techniques achieve the best results in smaller, earlier, precollapse lesions, while larger, more advanced, collapsed lesions may necessitate THA [7, 10, 12].

Augmentation procedures of the CD and multiple drilling decompression with bone marrow grafting and calcium sulfate/phosphate injectable bioceramics are showing promise [32, 33]. Gangji et al. conducted a randomized control trial comparing CD (*n* = 11) versus CD with autologous bone marrow implantation (*n* = 13), with a follow-up of 60 months, finding a significant decrease in the progression to collapse and a reduction in pain [34]. Civinini et al. used autologous bone marrow implantation along with a bioceramic consisting of calcium sulphate and calcium phosphate to backfill the core tract from the CD (*n* = 37), finding only a 3.3% conversion to THA in precollapse lesions (8.1% overall) at a mean follow-up of 20.6 months [35]. However, cost, lengthening of operative duration, technical feasible, and longer term outcomes should be considered for these procedures.

There are several important limitations of this study. Despite longer follow-up compared to similar existing studies, this cohort analysis has a small sample size and further investigation with larger cohorts is necessary. Furthermore, there was no comparison group because all patients that presented with ONFH were treated similarly and there was no cohort of non-arthroscopically assisted core decompression. Finally, there are no patient-reported outcome measurements available. However, we believe that this study is contributing to the literature by providing the first report of mid-term follow-up in arthroscopically managed ONFH patients.

## Conclusions

In conclusion, this is the longest reported follow-up of arthroscopic-assisted management of ONFH with mid-term outcomes of an average of 7 years. Hip arthroscopy-assisted core decompression is a new, evolving, and promising surgical approach to address osteonecrosis with high survivability for Stage I (100%) and Stage IIa (75%) lesions, while a 100% of Stage IIb lesions progressed to THA. In addition, hip arthroscopy allows for the treatment of the entire hip joint by addressing concomitant intraarticular pathologies and alleviating mechanical symptoms. Further studies with long-term follow-up and larger sample sizes are needed to evaluate the role of arthroscopy in ONFH.

## Data Availability

The datasets used and/or analyzed during the current study are available from the corresponding author on reasonable request.

## Abbreviations

AVN: Avascular Necrosis
CD: Core decompression
IRB: Institutional review board
MRI: Magnetic resonance imaging
ONFH: Osteonecrosis of the Femoral Head
PRP: Platelet rich plasma
THA: Total hip arthroplasty
VAS: Visual analog scale
